# Parallel substrate supply and pH stabilization for optimal screening of *E. coli* with the membrane-based fed-batch shake flask

**DOI:** 10.1186/s12934-018-0917-8

**Published:** 2018-05-09

**Authors:** P. Philip, D. Kern, J. Goldmanns, F. Seiler, A. Schulte, T. Habicher, J. Büchs

**Affiliations:** AVT-Biochemical Engineering, RWTH Aachen University, Forckenbeckstraße 51, 52074 Aachen, Germany

**Keywords:** Fed-batch, Shake flask, pH, Osmolality, MOPS buffer, Secondary substrate limitation, Screening, RAMOS, *Escherichia coli*

## Abstract

**Background:**

Screening in the fed-batch operation mode is essential for biological cultivations facing challenges as oxygen limitation, osmotic inhibition, catabolite repression, substrate inhibition or overflow metabolism. As a screening tool on shake flask level, the membrane-based fed-batch shake flask was developed. While a controlled supply of a substrate was realized with the in-built membrane tip, the possibilities for replenishing nutrients and stabilizing pH values was not yet exploited. High buffer concentrations were initially used, shifting the medium osmolality out of the biological optimum. As the growth rate is predefined by the glucose release kinetics from the reservoir, the resulting medium acidification can be compensated with a controlled continuous supply of an alkaline compound. The focus of this research is to establish a simultaneous multi-component release of glucose and an alkaline compound from the reservoir to enable cultivations within the optimal physiological range of *Escherichia coli*.

**Results:**

In combination with the Respiratory Activity MOnitoring System, the membrane-based fed-batch shake flask enabled the detection of an ammonium limitation. The multi-component release of ammonium carbonate along with glucose from the reservoir resulted not only in the replenishment of the nitrogen source but also in the stabilization of the pH value in the culture medium. A biomass concentration up to 25 g/L was achieved, which is one of the highest values obtained so far to the best of the author’s knowledge with the utilization of a shake flask and a defined synthetic medium. Going a step further, the pH stabilization allowed the decrease of the required buffer amount to one-fourth establishing an optimal osmolality range for cultivation. As optimal physiological conditions were implemented with the multi-component release fed-batch cultivation, the supply of 0.2 g glucose in a 10 mL initial culture medium volume with 50 mM MOPS buffer resulted in a twofold higher biomass concentration than in a comparable batch cultivation.

**Conclusions:**

The newly introduced multi-component release with the membrane-based fed-batch shake flask serves a threefold purpose of replenishing depleted substrates in the culture medium, stabilizing the pH throughout the entire cultivation time and minimizing the necessary amount of buffer to maintain an optimal osmolality range. In comparison to a batch cultivation, these settings enable to achieve higher biomass and product concentrations.
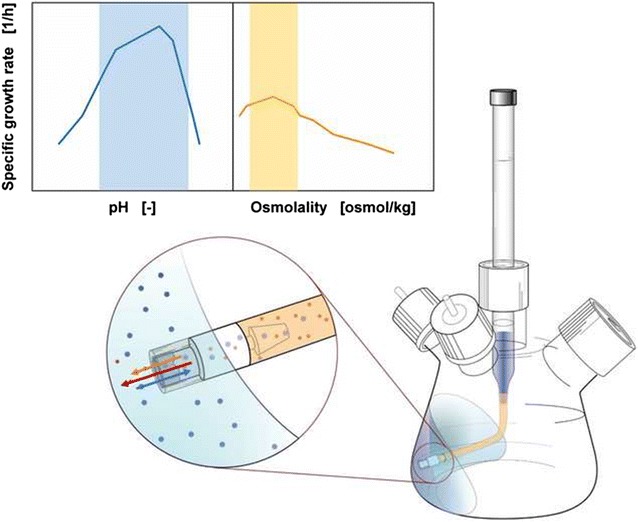

**Electronic supplementary material:**

The online version of this article (10.1186/s12934-018-0917-8) contains supplementary material, which is available to authorized users.

## Background

Initial bioprocess development involves a comprehensive screening program to identify the best suitable biological system in combination with feasible process parameters. Shaken bioreactors, in specific shake flasks, have become established screening tools to obtain process-relevant information [1, 2]. Since conventional shake flasks can only carry out batch cultivations, the membrane-based fed-batch shake flask was developed to allow fed-batch cultivations on small scale level [3]. In addition to implementing an identical operation mode to large scale production, a reliable screening tool is required to accurately reflect later cultivation conditions, having an impact on cell metabolism or viability. These parameters include amongst others pH and osmolality, which affect the growth rate of a microorganism. For *Escherichia coli* (*E. coli*), which is the most common prokaryotic production system [4], the optimal pH range is between 6.5 and 7.5, depending on the temperature [5]. During a cultivation, the pH is drifting as result of conversion and production of substrates and metabolic compounds. One such substrate is ammonium, which is a preferred nitrogen source supporting fast growth rates [6, 7]. Although the direct uptake of ammonium is possible through the transporter protein AmtB at very low ammonium levels or low pH environment [8], the usual uptake is through free diffusion of uncharged ammonia through the cell membrane [9, 10]. Hereby the pH-based conversion of ammonium to ammonia results in a free proton, which acidifies the surrounding culture medium [11].

An additional decrease of the pH in the culture medium is caused by overflow metabolites. Acetate is a common by-product of growth under anaerobic or oxygen-limiting conditions. But also under aerobic conditions, the presence of an excess amount of glucose can cause acetate production [12–14]. In this case, the carbon flux supply exceeds the demands and capacity of biosynthesis and energy generation within the central metabolism [15]. This results in an overloading of the tricarboxylic acid cycle and/or the electron transport chain, leading to an accumulation of acetate [13, 16]. High concentrations of acetate, around 5 g/L at pH 7, are supposed to reduce growth rates and biomass yields for *E. coli* cultivations [16, 17]. For various *E. coli* strains inhibition of growth through acetate has been described to result from acting as an uncoupler of the proton motor force or through the decrease of intracellular pH [12, 18]. Transport of acetate out of cells causes a decrease of pH in the culture medium. After depletion of glucose, consumption of acetate causes a pH increase again [19]. A further decrease in pH can occur from metabolically generated bicarbonate ions [20]. Hence, maintaining the physiological pH range of a microorganism requires a flexible response system. This requirement for shake flask cultivations has long been identified and several technical solutions have been developed.

A monitoring of pH is possible in shake flasks with a SENBIT measuring device (teleBITcom GmbH, Teltow/Germany) [21] or PreSens pH spots (PreSens Precision Sensing GmbH, Regensburg/Germany) [22], yet an additional pH controlling system is necessary to accomplish pH stabilization, e.g. the system presented by Weuster-Botz et al. [23] for shake flasks. With the help of pH probes as well as pumps and storage vessels for pH controlling agents, monitoring and titration to pre-set pH-values can be implemented in shake flasks. Yet, the required technical equipments, as well as their costs, to enable such a high flexibility of pH control limit the applicability of this system for high-throughput screening.

A further option to control pH is the polymer-based controlled release of sodium carbonate embedded in a silicon matrix, available as FeadBeads (Kühner, Birsfelden/Switzerland) [19]. An incremental release is achieved according to the number of beads used in the culture medium. For the initial stage of cultivations with low biomass concentrations, washing procedures of the utilized beads were required to limit the release in the beginning of cultivation. In general, the nearly linear release of sodium carbonate regardless of the progress of the cultured microorganism leads to a limited time frame of optimal pH values in the culture medium. This concept has to be seen as an open loop system without active pH control. A combined controlled release of glucose and a pH-stabilizing compound is in progress.

As an alternative approach, the EnBase medium (BioSilta Oy, Oulu/Finland), supplemented with complex medium additives and trace elements can be used [24]. The glucose release is accomplished with the degradation of starch with glucoamylase. Medium acidification can be compensated with the metabolism of complex compounds, e.g. yeast extract, protein peptones and casamino acids, which cause a pH increase. A drawback is that a significant metabolism of complex compounds takes place only in the absence of glucose, limiting the timeframe of pH stabilization.

Besides commercially available systems, several developments address the issue of pH stabilization in fed-batch shake flask cultivations based on varying functionalities. In the work of Weuster-Botz et al. shake flasks are equipped with pH probes for monitoring. Precise syringe pumps along with a substrate distribution system allowing both nutrient supply and pH control are utilized for this set-up [23]. Glucose-loaded hydrogels along with pH-managing hydrogels were used in the work of Sanil et al. to improve pH maintenance and higher biomass yields [25].

The easiest approach for a pH-controlled screening system on shake flask level is the usage of high buffer concentrations. In this study MOPS buffer is used for cultivations. It is a Good’s buffer, stabilizing the pH between 6 and 8 with a pK_a_ value at 7.044 at 37 °C [26]. A sufficient stabilization is accomplished with 200 mM MOPS for batch cultivations up to 20 g/L initial glucose concentration. The high buffer concentration comes at a cost of increased osmolality values [19]. This can significantly reduce the growth rate of microorganisms and impair the reliability of a screening process. In the work of Record et al. [27] an optimum growth at approximately 0.3 OsM and the growth rate approaching to zero at values between 1.7 and 2.0 OsM was shown for *E. coli* cultivations. In the work by Luchterhand et al. [28] calculations were presented showing that the combination of *E. coli* and mineral medium is a system with restrictive osmotic tolerance.

The focus of this research is to establish a multi-component release of glucose and an alkaline compound with the membrane-based fed-batch shake flask to remain within the optimal physiological range of *E. coli*. It is a standard shake flask mounted with a reservoir system as described by Bähr et al. and Philip et al. [3, 29] (see Additional file 1). The controlled release of substrate is enabled with a membrane tip along with an in-built membrane. The in-phase rotation of the membrane tip with the culture medium enables a continuous supply of substrate to cells. Furthermore, a continuous in-phase rotation ensures that the membrane tip does not act as a baffle. Thus, the membrane-based fed-batch shake flask can be compared to conventional batch shake flask cultivations, with the feeding equipment not negatively affecting the cultivation, as shown in the study of Bähr et al. [3]. The newly introduced multi-component release with the membrane-based fed-batch shake flask should serve a threefold purpose of replenishing depleted substrates in the culture medium, stabilizing the pH value throughout the entire cultivation time and minimizing the necessary buffer amount. With the last step, the osmolality can be shifted into an optimal range. The evaluation of these experiments was conducted with the Respiration Activity MOnitoring System (RAMOS) and optionally additional offline samples were taken for further analysis.

## Results and discussion

### Cultivations with increased glucose supply in batch and fed-batch cultivations

**Fig. 1:**
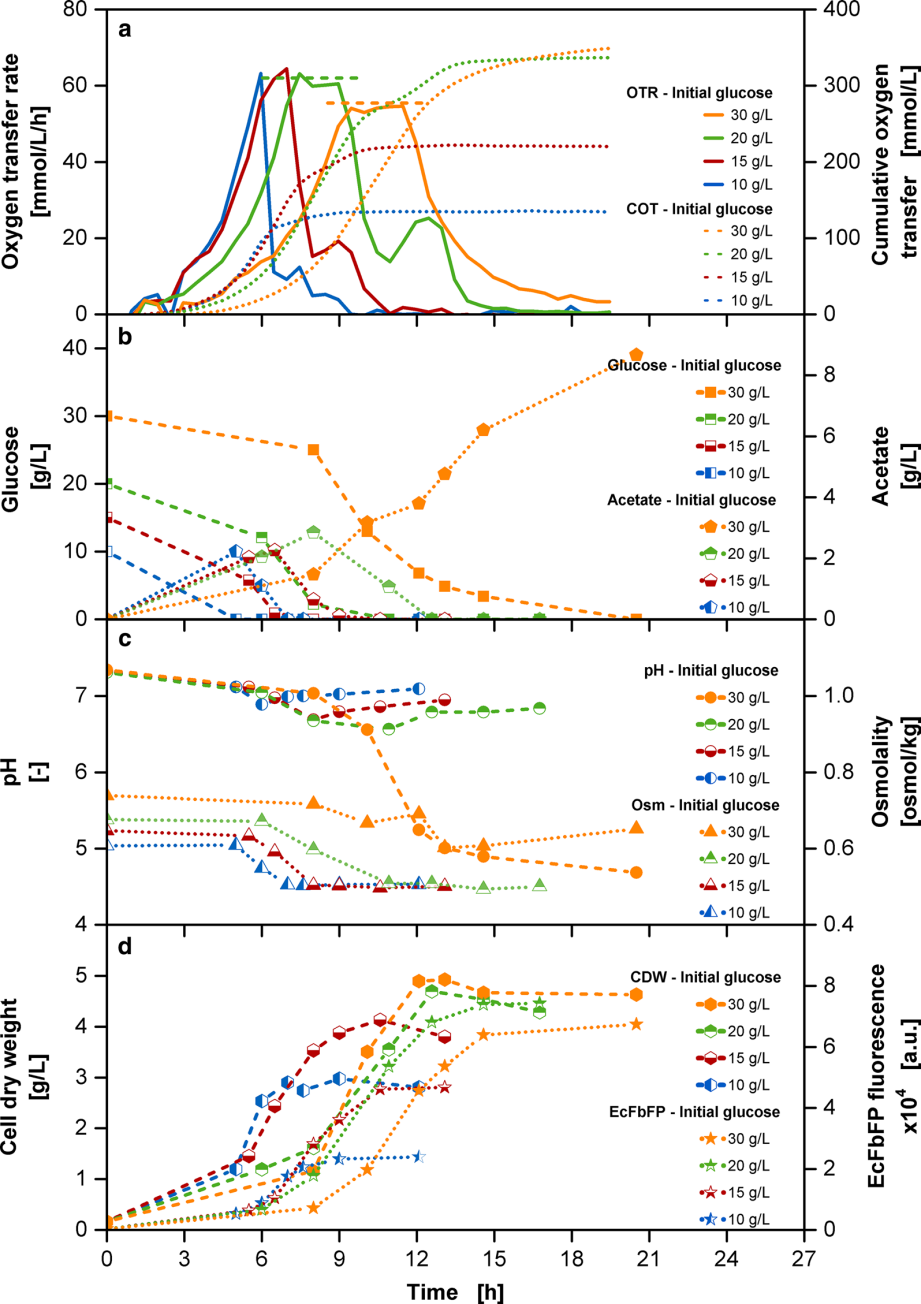
Variation of initial glucose concentration in batch cultivations. **a** Oxygen transfer rate (OTR) and cumulative oxygen transfer (COT). The dashed horizontal lines show calculated OTR_max_ levels [30] of 62.3 mmol/L/h for 20 g/L initial glucose concentration and 60.3 mmol/L/h for 30 g/L initial glucose concentration in the medium; **b** glucose concentration and acetate concentration; **c** pH and osmolality (Osm); **d** cell dry weight (CDW) and EcFbFP fluorescence; Cultivation conditions: Wilms-MOPS-mineral medium (initial glucose in culture medium: 10/15/20/30 g/L, 200 mM MOPS), temperature: 37 °C, shaking frequency: 350 rpm, shaking diameter: 50 mm, initial culture medium volume: 10 mL, inoculation OD_600_: 0.5; Strain: *E. coli* BL21 (DE3) pRhotHi-2-EcFbFP; Concentration values have been volume-corrected

The supply of high substrate concentrations in expectation of high biomass or product yields has been the motivation for many process developments. This undertaking with the batch operation mode is associated with many well-known challenges, summarized in Fig. 1. The initial glucose concentration was varied between 10 and 30 g/L. Figure 1a shows a clear prolongation of the lag phase of the oxygen transfer rate (OTR) and the cumulative oxygen transfer (COT) curves up to several hours with increasing initial glucose concentrations, causing alteration of the exponential phase as well. This is most likely the result from high osmolality values in the culture medium (Fig. 1c), impairing the growth of *E. coli*. Record et al. [27] describe the sudden change in osmolality in the environment of growing cells affecting e.g. cytoplasmic solute concentrations, turgor pressure and biosynthesis pathways. The exposure to high osmolalities occurs in the initial phase of cultivation, when cells from the pre-culture come into contact with a concentrated culture medium from the main culture. The growth rate was reduced from 0.7, 0.6, 0.5 to 0.4 1/h to for the batch cultivations with increasing glucose concentrations between 10 and 30 g/L in the culture medium. The COT progress is incremental with the initial glucose concentration and reaches a similar level for the 20 and 30 g/L glucose cultivations.

During the first OTR peak, glucose is consumed. Whereas the cultivations with 10–15 g/L glucose cultivations show a pointed peak, OTR plateaus appear in cultivations from 20 g/L onwards. Calculations of the maximum oxygen transfer capacity (OTR_max_) according to Meier et al. [30] were conducted considering osmolality of the medium, shaking parameters and available oxygen content. The calculated OTR_max_ of 62.3 mmol/L/h for 20 g/L initial glucose concentration and 60.3 mmol/L/h for 30 g/L initial glucose concentration agree well with the observed OTR plateaus, indicating that these cultivations are oxygen limited during these phases. The mandatory supply of sufficient oxygen is often an underestimated challenge, which when not ensured can lead to altered metabolism and affect cell viability [31].

Another common challenge is the production of overflow metabolites, like acetate with *E. coli* cultivations when glucose is present in excess [32]. Acetate is detectable in all tested cultivations (Fig. 1b). Its consumption is correlated with the second OTR peak. With 30 g/L glucose, only a single glucose peak occurs in the OTR, even though a significant amount of acetate of more than 8 g/L is present in the culture medium. Figure 1c shows a significant pH decrease down to values of around 4.6 for the cultivation with 30 g/L initial glucose, leading to the exhaustion of the buffer capacity and consequently detrimental conditions for cells.

An incremental increase in biomass and product concentrations can be seen in Fig. 1d. Whereas biomass concentration is in the same range for 20 and 30 g/L initial glucose concentration, the product amount of the 30 g/L initial glucose concentration is delayed and lower than the cultivation with 20 g/L initial glucose concentration. The inefficient conversion of a higher amount of substrate can be derived from the carbon flux supply exceeding the capacity of biosynthesis and energy generation within the central metabolism, causing an accumulation of acetate. With an increasing acetate accumulation, the buffer capacity of the culture medium is exceeded, moving the pH out of the optimal physiological pH range. The metabolism of the microorganisms is affected and its growth stagnates. Optimal cultivations under the given settings can thus be conducted only up to an initial glucose concentration of 20 g/L.

**Fig. 2:**
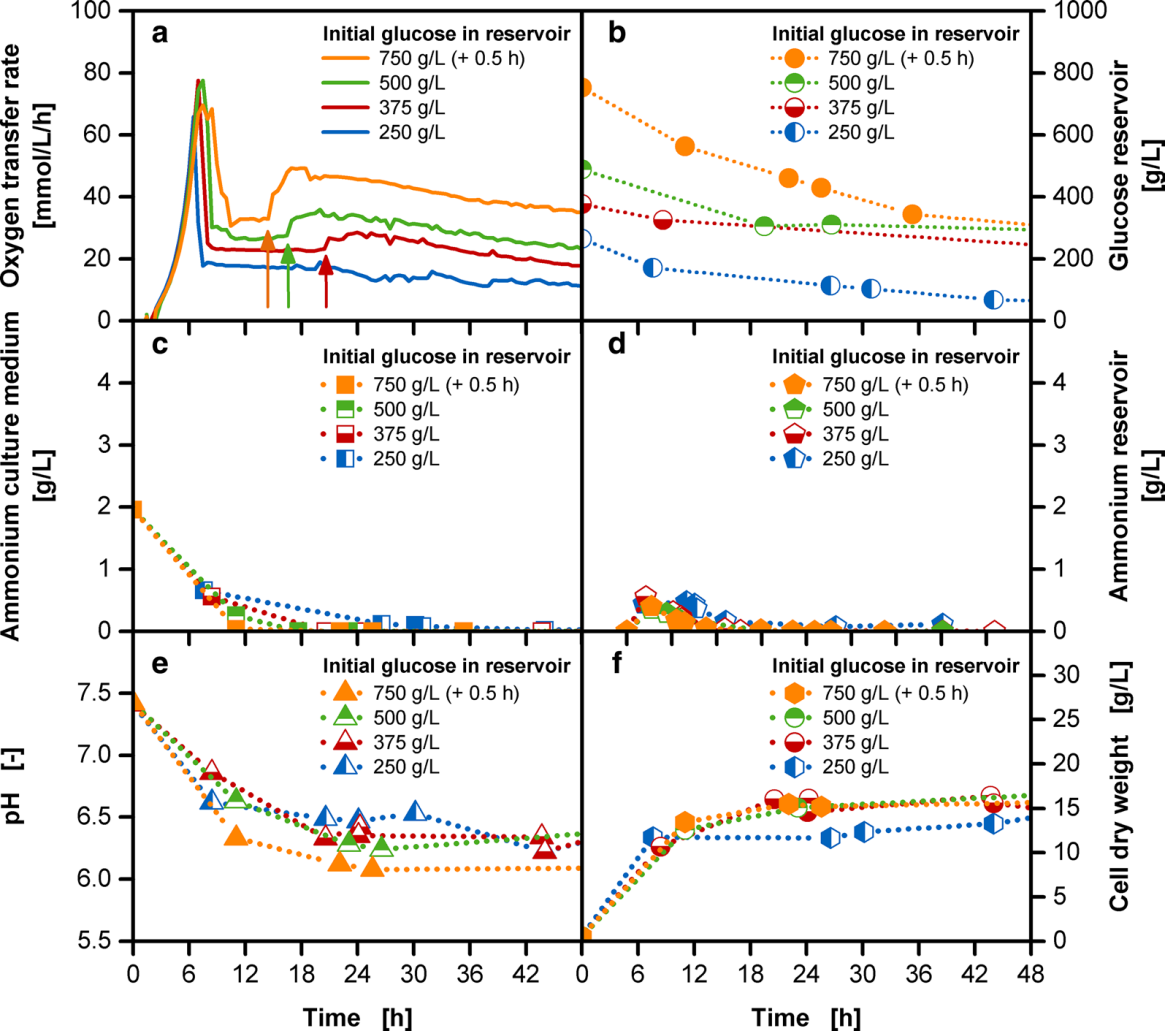
Characterization of abrupt OTR increases in the fed-batch phase at varied initial reservoir glucose concentrations with the membrane-based fed-batch shake flask. Depiction of **a** OTR; **b** glucose concentration in the culture medium; **c** ammonium concentration in the culture medium; **d** ammonium concentration in the reservoir; **e** pH in the culture medium; **f** cell dry weight; Cultivation conditions: Wilms-MOPS-mineral medium (culture medium: 200 mM MOPS with 7 g/L initial ammonium sulfate, initial reservoir glucose concentrations: 250/375/500/750 g/L, blue dextran concentration: 1 g/L, temperature: 37 °C, shaking frequency: 350 rpm, shaking diameter: 50 mm, initial culture medium volume: 10 mL, inoculation OD_600_: 0.5, reservoir filling volume: 2 mL, dialysis membrane: Reichelt 10–20 kDa, membrane area: 18.1 mm^2^; Strain: *E. coli* BL21 (DE3) pRhotHi-2-EcFbFP; Concentration values have been volume-corrected; The arrows point to the occurrence of abrupt OTR increases

The fed-batch operation mode is hence the ideal method of choice to circumvent the challenges of oxygen limitation, overflow metabolism, substrate or osmotic inhibition and catabolite repression [33]. A controlled glucose supply can be accomplished with the membrane-based fed-batch shake flask [29]. Choosing an unnecessary low release rate, though, will result in a slow growth rate, long cultivation time and low productivity. Thus, the challenge is to realize a sufficiently high glucose release while avoiding overflow metabolism and oxygen limited conditions [34]. In Fig. 2, a fed-batch fermentation with a variation of initial glucose concentrations in the reservoir between 250 and 750 g/L is shown. The batch phase is similar for all cultivations with a growth rate of 0.6 1/h for all tested reservoir compositions.

The increasing glucose release (Fig. 2b) with increasing glucose concentrations in the reservoir are reflected in elevated OTR levels of the fed-batch phase after the initial peak (Fig. 2a). The glucose release rates based on linear fits of data provided in Additional file 2 resulted in approximate release rates of 0.02, 0.015, 0.01 and 0.008 g/h for cultivations from 750 to 250 g/L initial glucose concentration in the reservoir, respectively. This is a result of an increased glucose concentration gradient between the reservoir and the culture medium. However, instead of a continuous horizontal progress in the fed-batch phase, abrupt OTR increases are detected at approximately 20.5, 16.5 and 14.5 h in cultivations with 375, 500 and 750 g/L initial glucose concentrations, respectively. The increased oxygen consumption is not a consequence of a sudden higher release of glucose as no accumulated amount of glucose was detected in the culture medium at these specific time periods (data not shown). Membrane leakages or measurement outliers can be omitted as possible causes, as a systematic occurrence of the abrupt OTR increase could be observed. With increasing initial reservoir glucose concentrations, the abrupt OTR increases appeared earlier and were steeper in height. As full functionality of the reservoir and measurement system was given, the fundamental cause for the abrupt OTR increases result from the biological cultivation itself.

Simple stoichiometric balances were calculated considering the growth of the microorganism on a substrate. These calculations showed that for cultivations with initial glucose concentrations from 375 g/L onwards, ammonia was depleted in the culture medium at approximately the same time point as the occurrence of the abrupt OTR increase (see Additional file 3). Offline measurements of ammonia verified the occurrence of an ammonia depletion (Fig. 2c). The 7 g/L ammonium sulfate initially provided to the culture medium is equivalent to approximately 1.9 g/L ammonium. As shown in the study of Philip et al. [29] and Fig. 2d the ammonia depletion is not caused by the reservoir acting as nutrient sink, as the initially accumulated amount of ammonium in the reservoir diffuses back upon the low concentration of ammonium in the culture medium. For the 250 g/L glucose cultivation, a depletion of ammonia was predicted from the stoichiometry, yet no abrupt OTR increase was observed. As Fig. 2f shows the biomass concentration is lower for the 250 g/L initial glucose cultivation than the rest of the cultivations with higher initial glucose concentration. Yet, as the ammonium concentration reaches critical values, a sufficient supply of secondary substrate should also be considered for cultivations with low initial reservoir concentrations as 250 g/L. In the cultivations with 350–750 g/L initial glucose concentration in the reservoir, the insufficient nitrogen supply most likely limits cell growth causing the appearance of an early stationary phase (Fig. 2f) [35]. As growth stagnates at this point of time the appearance of concentration plateaus can be observed for the glucose concentrations in the reservoir (Fig. 2b), as glucose is not being consumed with the initially present rate anymore. With the help of the stoichiometric balance theoretical values for glucose consumption, glucose accumulation and production of biomass were calculated and are depicted in Additional file 3. The calculated values show the potential cultivation progress with a constant substrate release rate. Approximately 2.3 mol O_2_/mol glucose are required for the cultivation of a growing microorganism. When growth is not possible, glucose combustion takes place to enable a sufficient energy supply. A certain amount of the released glucose is combusted requiring a higher oxygen amount, noticeable in the spontaneous OTR increase. A similar dissipation process to carbon dioxide and heat is described by El-Mansi et al. [13] as one of the response possibilities, when the carbon flux exceeds the metabolic capacity of the central pathways. As a higher turnover of oxygen is seen at the time points of abrupt OTR increases, this explanation in terms of a combustion reaction is most likely applicable and requires further analysis for verification. Noteworthy though is the easy identification of a secondary substrate limitation caused by ammonium with the simple combination of the membrane-based fed-batch shake flask with the RAMOS device. No offline sampling and analysis is required, as only the abrupt OTR increase needs to be detected online.

To counteract the waste of valuable glucose as substrate, a replenishing of ammonium is essential. Further, Fig. 2e shows that with increasing glucose concentrations in the reservoir the buffer capacity of MOPS is close to exhaustion reaching pH values of around pH 6. In comparison to Fig. 1 the overall pH progress and the exploitation of the capacity of the buffer varies between the batch and the fed-batch operation mode. As the fed-batch phase results from glucose limitation, the concentration of measurable glucose is low in the culture medium, hence, reducing overflow metabolites and a strong pH drift as seen in the 30 g/L batch cultivation. Still, an increased ammonium supply and pH stabilization need to be implemented for improved fed-batch cultivations.

### Simultaneous implementation of secondary substrate supply and pH optimization

**Fig. 3:**
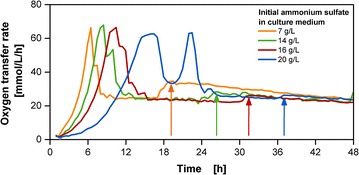
Oxygen transfer rate of cultivations with increased ammonium sulfate concentrations in the culture medium in cultivations with the membrane-based fed-batch shake flask. Cultivation conditions: Wilms-MOPS-mineral medium (culture medium: 200 mM MOPS with varied initial ammonium sulfate, initial reservoir glucose concentrations: 500 g/L, blue dextran concentration: 2 g/L), temperature: 37 °C, shaking frequency: 350 rpm, shaking diameter: 50 mm, initial culture medium volume: 10 mL, inoculation OD_600_: 0.5, reservoir filling volume: 2 mL, dialysis membrane: Reichelt 10–20 kDa, membrane area: 18.1 mm^2^; Strain: *E. coli* BL21 (DE3) pRhotHi-2-EcFbFP; The arrows point to the occurrence of abrupt OTR increases

The identified depletion of ammonium can be overcome by increasing the initial amount of 7 g/L ammonium sulfate supplied in the culture medium (Fig. 3). With increasing ammonium sulfate concentrations, the abrupt OTR increase is delayed and is at a lower amplitude. However, a significant prolongation of the lag phase and a decrease of growth rate from approximately 0.6, 0.5, 0.4 and 0.3 1/h can be observed for increasing ammonium sulfate concentrations in the medium from 7, 14, 16 and 20 g/L, respectively. The delay in growth results in a higher initial accumulation of glucose, leading to oxygen limitation and increased acetate production indicated by the increased second peak. Müller et al. [36] describe that growth impairment resulting from the addition of ammonium sulfate is a result of enhanced osmolality of the medium. For the concentration of 7, 14, 16 and 20 g/L the initial osmolality values were 0.58, 0.65, 0.72, and 0.80 osmol/kg, respectively. From approximately 3 g/L ammonia concentration, which is already exceeded with 14 g/L ammonium sulfate, ammonium toxicity on growth needs to be considered [4]. According to literature, this critical threshold may vary with the specific biological system and medium composition [36]. The progressive inhibition with increasing ammonium concentration has been observed for cultivations with various organisms as *Corynebacterium glutamicum*, *E. coli* and *Bacillus subtilis* [36]. For *Saccharomyces cerevisiae,* this resulted in a 50% reduced biomass concentration [37]. Hence, the supply of ammonium with increased ammonium sulfate levels in the culture medium should be avoided to evade inhibiting or toxic effects. In addition, higher ammonium sulfate concentrations cause a stronger decrease of pH in the culture medium, which moves the pH even further away from the biological optimum.

**Fig. 4:**
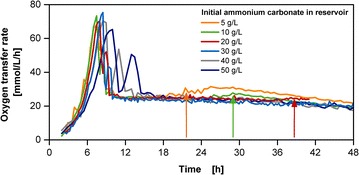
Screening for adequate ammonium carbonate concentrations in the reservoir for the multi-component release with the membrane-based fed-batch shake flask. Cultivation conditions: Wilms-MOPS-mineral medium (culture medium: 200 mM MOPS with 7 g/L initial ammonium sulfate, initial reservoir glucose concentrations: 500 g/L with varied initial ammonium carbonate in the reservoir along with 17.14 g/L K_2_HPO_4_, blue dextran concentration: 2 g/L), temperature: 37 °C, shaking frequency: 350 rpm, shaking diameter: 50 mm, initial culture medium volume: 10 mL, inoculation OD_600_: 0.5, reservoir filling volume: 2 mL, dialysis membrane: Reichelt 10–20 kDa, membrane area: 18.1 mm^2^; Strain: *E. coli* BL21 (DE3) pRhotHi-2-EcFbFP; The arrows point to the occurrence of abrupt OTR increases

The membrane-based fed-batch shake flask offers the possibility to supply the necessary amount of additional ammonium through the reservoir system in a concentration range in the culture medium, which is not inhibiting or toxic to cells. The release of ammonium carbonate from the reservoir enables the supply of ammonium and stabilization of pH. Figure 4 shows the coupled release of 500 g/L initial glucose concentration in the reservoir with varied ammonium carbonate concentrations between 5 and 50 g/L. The first OTR peak results from the consumption of glucose, the second from acetate consumption (Fig. 1) and the abrupt OTR increases marked with arrows indicate an underlying ammonium depletion (Fig. 2). With increasing ammonium salt concentration, a slight diversification of the OTR during the exponential phase is observed with growth rates varying between 0.4 and 0.5 1/h, as well as a more dominant acetate peak. These effects most likely result from a slightly increased osmolality in the initial phase with low biomass concentrations. Noteworthy though is the successful avoidance of abrupt OTR increases in the fed-batch phase. With increasing ammonium carbonate concentration in the reservoir this phenomenon is delayed in its appearance at approximately 21.5, 29, 39 h for 5, 10, 20 g/L ammonium carbonate concentration respectively, as well as reduced in its amplitude. The abrupt OTR increase is non-existent from above the concentration of 30 g/L ammonium carbonate. As the OTR progress of the culture with 30 and 40 g/L ammonium carbonate is similar, the latter was used for further experimentation to accomplish improved pH stabilization. The screening experiments presented in Figs. 3 and 4 demonstrate the simple approach of acquiring relevant information in a fast manner without tedious offline sampling and analysis. With this procedure of screening the identification and avoidance of an ammonia limitation was accomplished with just online monitoring of OTR. When a detailed characterization is required, the screening can be extended with offline sampling.

**Fig. 5:**
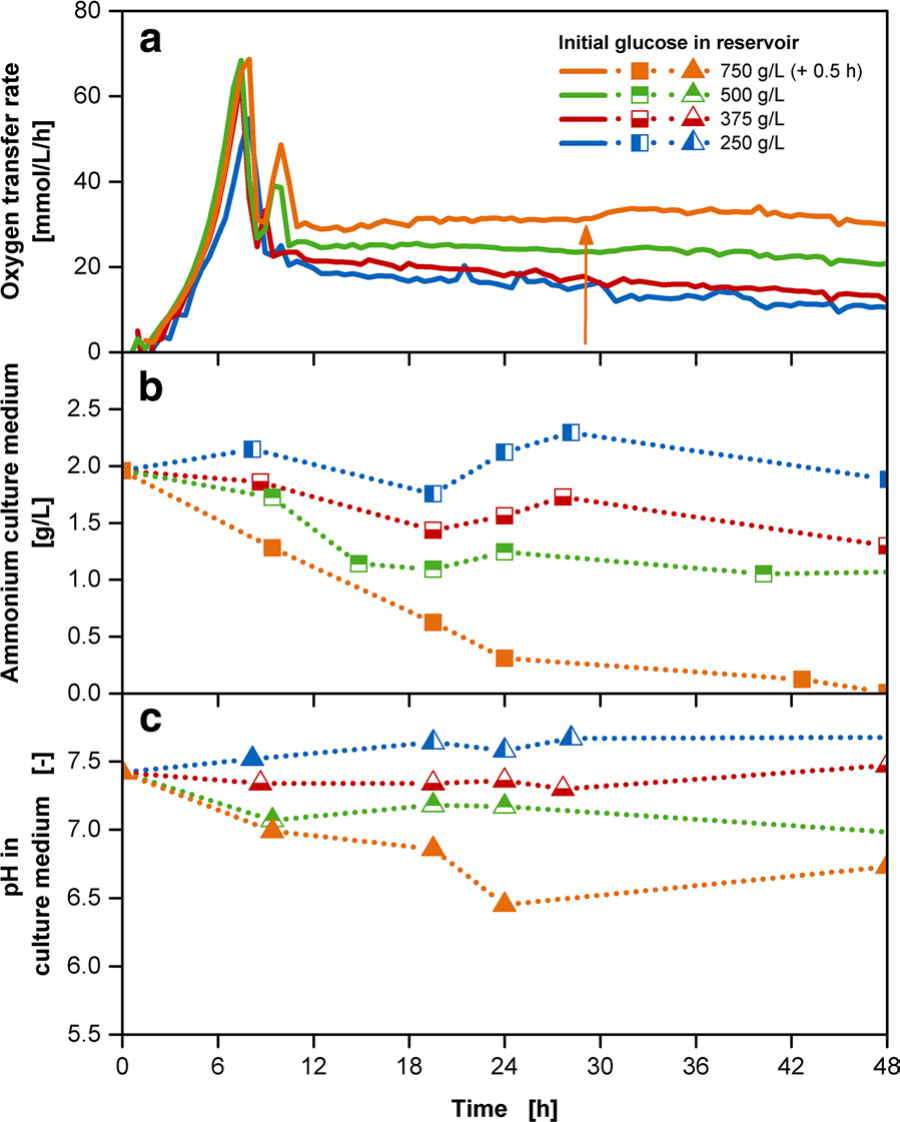
Characterization of the multi-component fed-batch cultivations with varied initial glucose concentrations and 40 g/L ammonium carbonate in the reservoir with the membrane-based fed-batch shake flask. Depiction of **a** oxygen transfer rate; **b** ammonium concentration in the culture medium; **c** pH in the culture medium; Cultivation conditions: Wilms-MOPS-mineral medium (culture medium: 200 mM MOPS with 7 g/L initial ammonium sulfate, initial reservoir glucose concentrations: 250/375/500/750 g/L, 40 g/L ammonium carbonate, blue dextran concentration: 1 g/L), temperature: 37 °C, shaking frequency: 350 rpm, shaking diameter: 50 mm, initial culture medium volume: 10 mL, inoculation OD_600_: 0.5, reservoir filling volume: 2 mL, dialysis membrane: Reichelt 10–20 kDa, membrane area: 18.1 mm^2^; Strain: *E. coli* BL21 (DE3) pRhotHi-2-EcFbFP; Concentration values have been volume-corrected; The arrow points to the occurrence of an abrupt OTR increase

Offline experiments with the simultaneous release of glucose with concentrations in the reservoir in the range between 250–750 g/L and 40 g/L ammonium carbonate were conducted for the time-resolved analysis of ammonium concentrations and pH values (Fig. 5). With increasing initial glucose concentrations in the reservoir with a growth rate of approximately 0.5 1/h, the resulting OTR curves show a similar batch phase, yet incremental acetate peaks as well as OTR levels of the fed-batch phase (Fig. 5a). For cultivations, up to 500 g/L initial glucose concentration in the reservoir, the abrupt OTR increase in the fed-batch phase is non-existent. The ammonium remains in the range of the initially provided amount of 1.9 g/L for the cultivation with 250 g/L initial glucose concentration in the reservoir (Fig. 5b). A decrease in the ammonium concentration can be noted for the cultivations with a glucose reservoir concentration of 375 and 500 g/L, but remains above zero throughout the entire cultivation time. A specific adaption of the ammonium concentration in the reservoir to each individual glucose release is not required. Additional file 4 shows that ammonium is still present in the reservoir during the cultivation period of 48 h for all conducted cultivations.

For cultivation with 750 g/L initial glucose concentration, the ammonium concentration in the culture medium comes close to depletion and a minor OTR increase in the fed-batch phase around 28 h can be observed (see Additional file 5). The underlying cause can either be a limiting substance other than ammonium or the release from the reservoir is not fast enough to compensate the consumption through the culture. The abrupt OTR increase could be delayed and reduced with increasing ammonium salt concentration up to 65 g/L (see Additional file 6).

As mentioned earlier, pH stabilization is the second requirement for optimal fed-batch cultivations. The ammonium carbonate addition is able to compensate the ongoing acidification resulting from the proton release with the ammonium uptake [20]. Figure 5c shows for cultivations with initial glucose concentrations in the reservoir between 250 and 500 g/L the pH value remaining between 7.0 and 7.5 throughout the entire cultivation time. A decrease can be noted for the cultivation with 750 g/L initial glucose concentration. Yet, the pH value is still in the acceptable range well above the critical value of 5.8 mentioned by Scheidle et al. [19].

The membrane-based fed-batch shake flask enables the implementation of a self-regulatory system resulting from the controlled release of glucose with the release of pH-stabilizing salt adapted to the metabolic activity of the cultured microorganism dictated by the fed-batch operation mode. The term self-regulatory system refers to a defined or regulated metabolic state as a result of a continuous supply of a controlled amount of substrate. Independent of varying inoculum concentrations, time points of inoculation and other factors contributing to a divergence of lag and batch phases, the biomass will eventually reach the carbon source limited state. From this time on, the cells will grow as fast as the supply of carbon source allows. This will result in an equalization of biomass concentrations and growth rates, which are accomplished dependent on a pre-defined glucose release kinetic [38]. Because of this self-regulating effect the increase in biomass, the resulting acidification and the required amount of alkaline compound can be correlated. In contrast, when the supply of substrate and pH-stabilizing compound are not coupled, an adequate pH stabilization is challenging. In the study of Scheidle et al. [19], it was shown that at initially low biomass concentration the release of base caused a pH shift to high values, as medium acidification was over-compensated. Then followed a cultivation period where at increased biomass concentration the base release adequately compensated acidification. But, as biomass growth stagnated, a surplus of base resulted in a shift to higher pH values. Hence the optimal operation window is very small. When supplying both, substrate and the base in the fed-batch operation mode, the previously non-regulated base supply can then be tuned to the substrate release so that the entire cultivation becomes self-regulated.

**Fig. 6:**
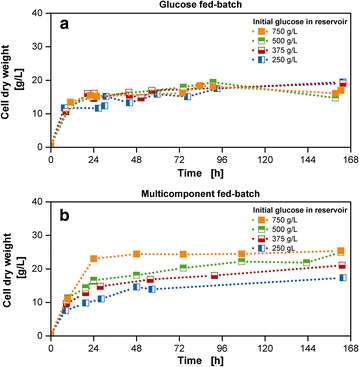
Cell dry weight results for fed-batch cultivations with varied reservoir compositions. **a** Biomass concentrations with glucose fed-batch cultivations; **b** biomass concentrations with glucose fed-batch cultivations along with 40 g/L ammonium carbonate; all measured CDW values have been volume-corrected; Cultivation conditions: Wilms-MOPS-mineral medium (culture medium: 200 mM MOPS with 7 g/L initial ammonium sulfate, initial reservoir glucose concentrations: 250/375/500/750 g/L, blue dextran concentration: 1 g/L), temperature: 37 °C, shaking frequency: 350 rpm, shaking diameter: 50 mm, initial culture medium volume: 10 mL, inoculation OD600: 0.5, reservoir filling volume: 2 mL, dialysis membrane: Reichelt 10–20 kDa, membrane area: 18.1 mm^2^; Strain: *E. coli* BL21 (DE3) pRhotHi-2-EcFbFP; Concentration values have been volume-corrected

The benefit of the multi-component fed-batch cultivation becomes obvious when considering the resulting cell dry weight values. In comparison to fed-batch cultivations with only glucose release (Fig. 6a), the simultaneous release of glucose and ammonium carbonate (Fig. 6b) increased the biomass concentration from approximately 20 to 25 g/L. According to the best of the author’s knowledge, this is one of the highest values achieved so far for shake flasks with standard geometry in combination with a mineral medium. Hereby the values have been corrected with factors considering evaporation and water-back-diffusion [29]. This correction is compulsory as otherwise concentrating effects in the culture medium falsify the results up to a factor of almost five (See Additional file 7). With the use of specialized shake flask geometries, enhancing oxygen transfer and nutrient boosters containing complex compounds, values up to OD 73–78, corresponding to cell dry weight values of 19.7–21.1 g/L were received for *E. coli* cultivations in the study of Ukkonen et al. [39]. The requirement of manual addition of glucose polysaccharides at different time-points, as well as enzyme and nutrient boosters increase handling efforts. This is simplified with the membrane-based fed-batch shake flask, which enables the simultaneous release of several compounds without the necessity of stopping the shaker and risking sterility by opening the culture vessel. A further benefit is the usage of only mineral medium components, ensuring a higher probability for reproducible cultivations [40]. Biomass concentrations higher than 25 g/L can possibly be achieved by increasing oxygen supply or addition of trace element solution, containing selenium, nickel and molybdenum [41]. The release of the trace elements from the reservoir system is advantageous as these compounds are toxic in higher initial concentrations in the culture medium or may cause precipitation in reaction with other medium compounds [42].

### Challenges and opportunities with the multi-component release

When working with highly concentrated reservoir solutions several challenges may arise. This was closely analyzed in non-biological experiments with various reservoir compositions.

**Fig. 7:**
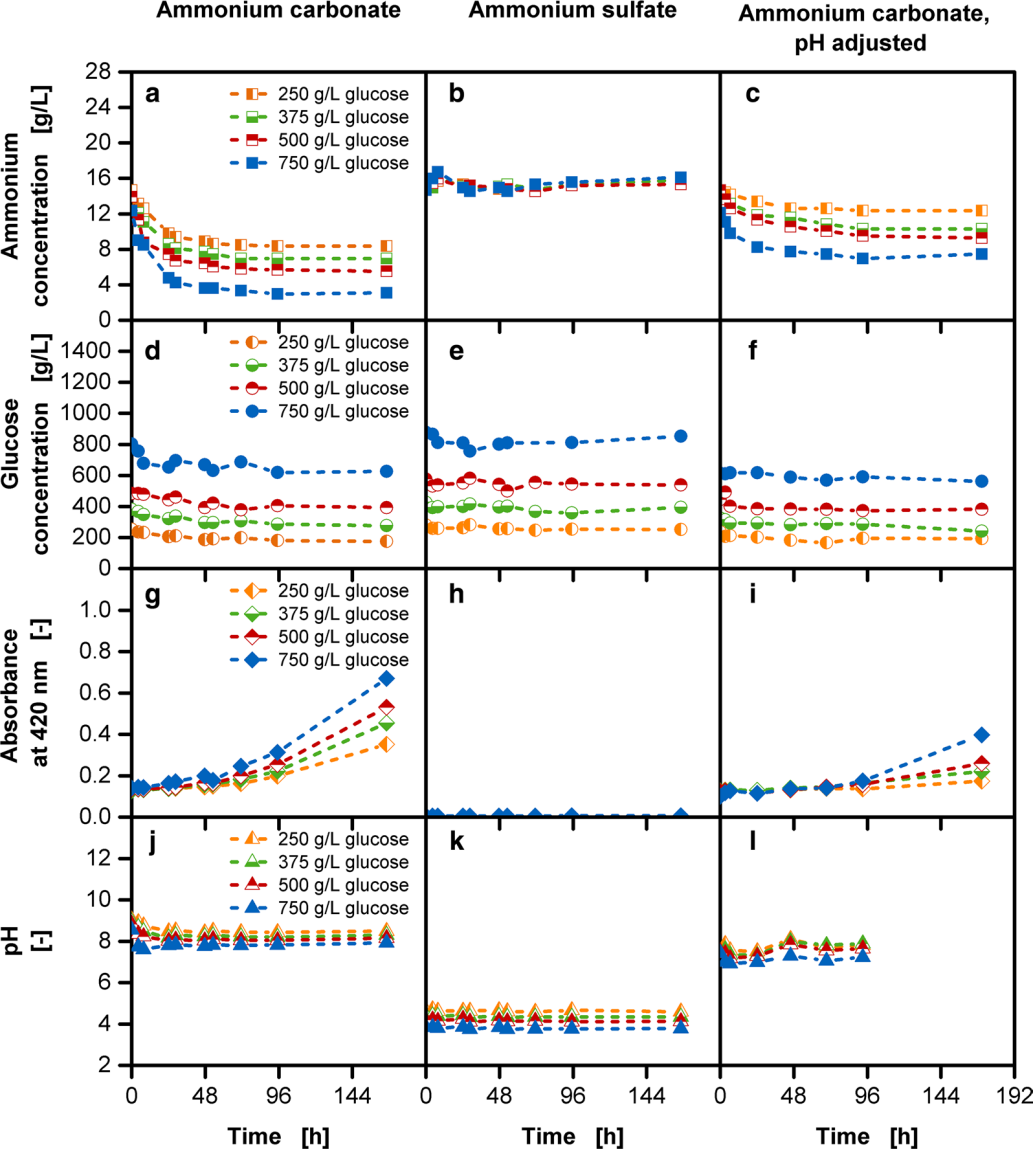
Long-term analysis of various reservoir compositions without biology. Left column: Reservoir composition with various initial glucose concentrations and 40 g/L ammonium carbonate, depiction of **a** ammonium concentration; **d** glucose concentration; **g** absorbance measurement to characterize Maillard reaction compounds; **j** pH; middle column: reservoir composition with various initial glucose concentrations and 50.47 g/L ammonium sulfate, depiction of **b** ammonium concentration; **e** glucose concentration; **h** absorbance measurement; **k** pH; right column: reservoir composition with various initial glucose concentrations with 40 g/L ammonium carbonate and initial pH adjusted to 7.5 with sulfuric acid, depiction of **c** ammonium concentration; **f** glucose concentration; **i** absorbance measurement; **l** pH; conditions: temperature: 37 °C, volume: 45 mL, initial glucose concentration: 250/375/500/750 g/L, blue dextran concentration: 1 g/L

A high amount of ammonium carbonate causes an increase of the pH value in the reservoir solution. This can lead to the outgassing of ammonia starting at one unit lower than pK_a_ value of 9.25. Further, the presence of high glucose and ammonium concentration can cause side-reactions to take place, resulting in a variety of unwanted reaction compounds [43]. Factors influencing the progress of side-reactions categorized as Maillard reaction are temperature, initial glucose concentration and catalyst loading [44]. Maillard reaction compounds can be detected with a 420 nm absorbance measurement [45, 46]. In the first column of Fig. 7 the analysis of the reservoir composition with varied initial glucose concentrations along with 40 g/L ammonium carbonate is shown. With increasing initial glucose concentrations, a decrease of ammonium (Fig. 7a) and glucose (Fig. 7d) in the initial phase is observed reaching an almost constant level after approximately 48 h. The initial pH values are close to the pK_a_ value of ammonia (Fig. 7j) possibly causing its outgassing. Further, the absorbance measurement at 420 nm shows an increase of the signal after approximately 48 h (Fig. 7g), although no significant changes are observed in the ammonium concentration in this period (Fig. 7a). As the Maillard reaction is slow under the given settings the compounds may be detectable only after a certain time.

In the second set-up, the tested reservoir compositions contain varying glucose concentrations and ammonium sulfate concentrations instead of ammonium carbonate. The ammonium sulfate concentration was chosen in such a way that it corresponds to the initially provided ammonium concentration with 40 g/L ammonium carbonate. An almost constant ammonium (Fig. 7b) and glucose (Fig. 7e) concentration can be observed throughout the entire experimental time. Low pH values (Fig. 7k) between 3.8 and 4.6 are measured in the reservoir and no signal for the 420 nm absorbance measurement (Fig. 7h) was detected throughout the entire experimental time. This reservoir composition is suitable for cultivations requiring only additional ammonium supply without pH stabilization.

When high glucose and ammonium concentrations along with pH stabilization is required, the loss of ammonium with the usage of ammonium carbonate in the reservoir can be circumvented by decreasing the initial pH value to 7.5 in the reservoir (Fig. 7l). As an acidic compound, sulfuric acid was used for pH adjustment. Figure 7c, f show that the initial decrease of ammonium and glucose is reduced in comparison to Fig. 7a, d. The signal of the 420 nm absorbance measurement (Fig. 7i) is constant for almost 96 h and the final values are lower in comparison to Fig. 7g. pH values in the reservoir remain below the critical pH limit of 9.25 (Fig. 7l).

### Simultaneous implementation of secondary substrate supply and optimization of pH and osmolality range

**Fig. 8:**
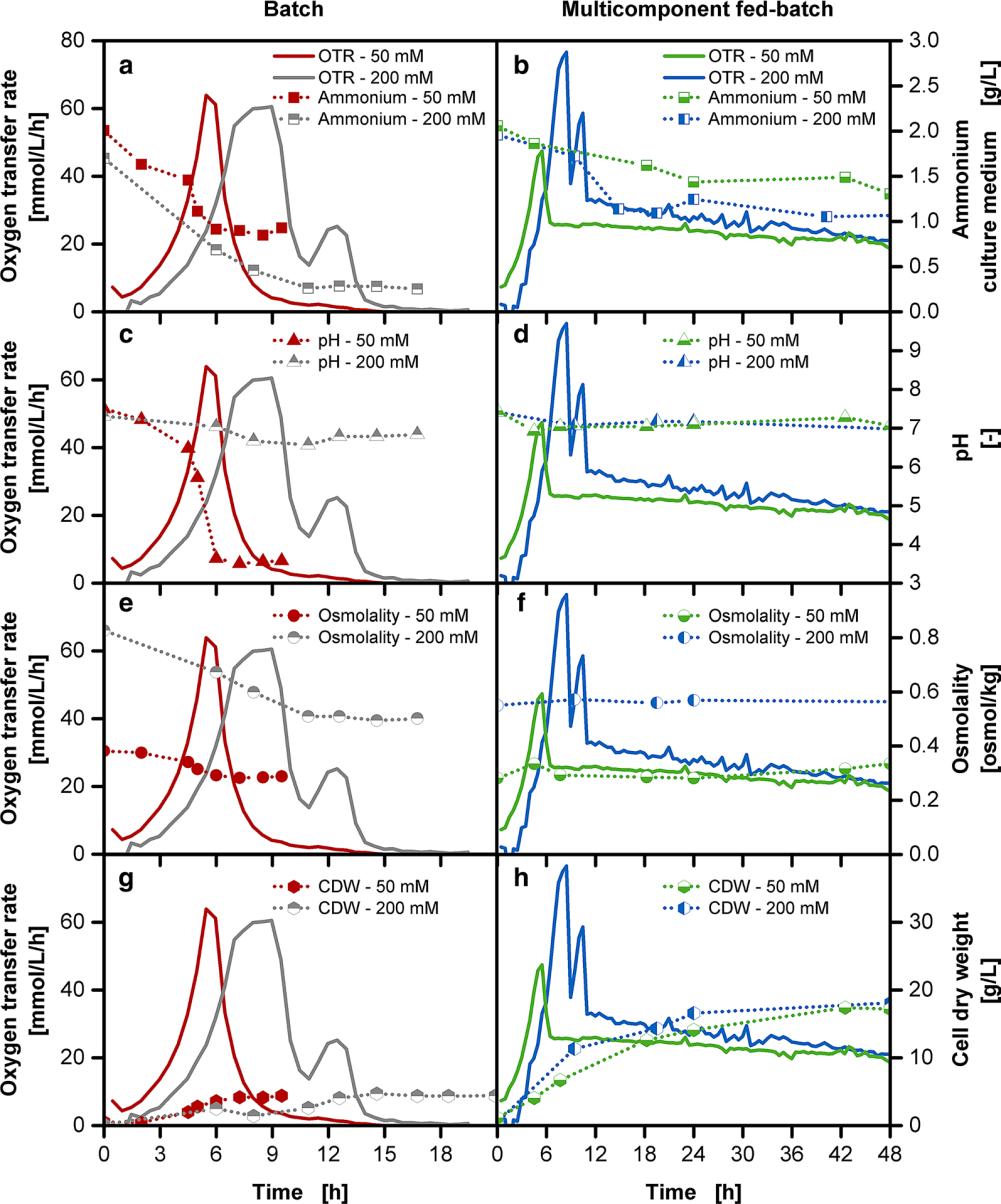
Characterization of batch and multi-component fed-batch cultivations with standard and reduced buffer concentration with *E. coli* BL21 (DE3) pRhotHi-2-EcFbFP. Left column: Depiction of **a** oxygen transfer rate (OTR) and ammonium; **c** pH in the culture medium; **e** osmolality; **g** Cell dry weight (CDW) of batch cultivations with Wilms-MOPS-mineral medium, with 200 and 50 mM MOPS buffer, 20 g/L glucose and inoculation OD_600_: 0.5; right column: depiction of **b** oxygen transfer rate (OTR) and ammonium; **d** pH in the culture medium; **f** osmolality; **h** cell dry weight (CDW) of fed-batch cultivations with Wilms-MOPS-mineral medium with 200 mM MOPS buffer, 500 g/L initial glucose concentration, 40 g/L ammonium carbonate, blue dextran concentration: 1 g/L in the reservoir or 50 mM MOPS buffer with 500 g/L initial glucose concentration, 60 g/L ammonium carbonate, blue dextran concentration: 1 g/L in the reservoir, temperature: 37 °C, shaking frequency: 350 rpm, shaking diameter: 50 mm, initial culture medium volume: 10 mL, inoculation OD_600_: 0.5, reservoir filling volume: 2 mL, dialysis membrane: Reichelt 10–20 kDa, membrane area: 18.1 mm^2^; strain: *E. coli* BL21 (DE3) pRhotHi-2-EcFbFP; Concentration values have been volume-corrected

In the previously described experiments with the multi-component release, the pH stabilization is based on the MOPS buffer provided initially in the culture medium, complemented with the ammonium carbonate release from the reservoir. A major drawback is still the high amount of required buffer resulting in a non-optimal high osmotic pressure. To implement optimal physiological cultivation conditions for *E. coli*, the reduction of the buffer concentration is an essential step. Figure 8 compares the impact of standard cultivations with 200 mM MOPS and a reduced buffer concentration of 50 mM MOPS, for both batch and fed-batch cultivations. The concentration of 50 mM MOPS buffer was chosen as it allows an osmolality value of 0.4 osmol/kg close to the optimal range for *E. coli* in comparison to the osmolality of 0.8 osmol/kg for the 200 mM MOPS buffer cultivation in batch operation mode (Fig. 8e) [27]. The standard batch cultivation with 200 mM MOPS for 20 g/L initial glucose amount (Fig. 8a) shows the typical progress of an initial high OTR peak resulting from the glucose metabolism with a growth rate of 0.57 1/h in the exponential phase followed by a second peak due to the consumption of the overflow metabolite acetate. The initially supplied amount of ammonium is almost depleted at the end of the cultivation (Fig. 8a). In contrast, the cultivation with 50 mM MOPS buffer shows a shorter lag-phase due to the reduced osmotic pressure with a slightly increased growth rate of 0.6 1/h and only a single peak. The residual amount of ammonium (Fig. 8a) and the lower integral area under the OTR curve already point to the fact that an incomplete conversion of glucose has taken place, with around 6–7 g/L glucose in the final samples of the culture medium (data not shown). Whereas the pH (Fig. 8c) remains almost constant at values around pH 7 with the 200 mM MOPS buffer cultivation, a drastic pH drop below pH 4 can be observed for the 50 mM MOPS cultivation. Previous studies with *E. coli* showed that below pH 5.8 cultivations are significantly impaired [19]. This compromise of either setting the pH or the osmolality to an optimal value does not need to be made with the multi-component release of the membrane-based fed-batch shake flask. The reduction of buffer in the fed-batch cultivations from 200 to 50 mM MOPS was compensated with an increase of ammonium carbonate from 40 to 60 g/L in the reservoir. The effect of buffer reduction becomes obvious with the reduced lag-phase, shorter accumulation of glucose and the absence of the acetate peak (Fig. 8b). The growth rate is at approximately 0.38 1/h with the 200 mM MOPS fed-batch cultivation and at approximately 0.43 1/h for the 50 mM fed-batch cultivation. The fed-batch OTR level of the 50 mM MOPS cultivation is slightly lower than the cultivation with 200 mM. The underlying cause could result from altered release rates due to changed osmolality ratios between culture medium and reservoir and require further analysis. The supply of 0.2 g total glucose in a 10 mL culture medium equivalent to 20 g/L with 50 mM MOPS buffer resulted in a twofold higher biomass concentration (Fig. 8h) than in the comparable 50 mM batch cultivation (Fig. 8g). As the batch phase contributes significantly to biomass production, a shorter batch phase results in slightly lower initial biomass concentration. But towards the end of the cultivation, similar biomass concentrations are reached in the multi-component release cultivations with the starting MOPS buffer concentration of 50 and 200 mM (Fig. 8h). This can possibly be caused by an unknown substrate limitation. Higher concentrations can possibly be obtained when the composition of *E. coli* and yield coefficients of the medium components are considered for an improved medium recipe [47–50]. The multi-component release allows the necessary supply of ammonium (Fig. 8b) and a comparable pH progress between the cultivations with the reduced buffer system and the standard buffer system (Fig. 8d). With the 50 mM MOPS buffer, the cultivation is conducted within the physiological osmolality optimum of 0.3 osmol/kg in comparison to the osmolality of 0.6 osmol/kg in the 200 mM MOPS buffer cultivation in the fed-batch operation mode (Fig. 8f) [27]. The stabilization of cultivation conditions allows for a higher conversion of glucose to higher biomass production compared to batch cultivations (Fig. 8g, h). A similar concentration range of biomass was obtained for the fed-batch cultivations with 200 and 50 mM MOPS, showing that the examined system *E. coli* BL21 (DE3) pRhotHi-2-EcFbFP on the long run seems to cope with suboptimal osmolality values.

## Conclusion

The multi-component release of the membrane-based fed-batch shake flask is a self-regulatory system resulting from the controlled release of glucose combined with the release of different components. This approach is of benefit to circumvent a secondary substrate limitation, as e.g. caused by ammonium. It was shown that an increase of the initial concentration of ammonium sulfate in the culture medium hampered metabolic activity of cultivations, causing amongst others prolonged lag-phases, overflow metabolites and oxygen limitations. The underlying cause is ascribed to elevated osmolality and toxicity of ammonium in the culture medium. Hence, the release of additional supplements from the reservoir is an optimal solution to avoid high initial concentrations. The reservoir composition can be extended to the release of multiple components to replenish the culture medium. The limiting factors for this strategy are the solubility of released compounds as well as possible side-reactions between the dissolved components in the reservoir. The addition of ammonium sulfate was shown to decrease the loss of ammonium and glucose to unwanted side-reactions.

As the growth rate is predefined by the glucose release kinetics, the resulting acidification and the required amount of alkaline component is known. In this study, it was shown that a detailed characterization of metabolic pathways and stoichiometry to quantify the acidification is not necessary. Through a simple screening experiment, the supply of 40 g/L ammonium carbonate in the reservoir was identified to stabilize the pH for the release of glucose up to a concentration of 500 g/L initial concentration. The pH drift as result of conversion of substrates and production of metabolic compounds was sufficiently compensated.

The presented multi-component release with the membrane-based fed-batch shake flask offers the possibility of a customized reservoir composition to achieve high biomass or product yields with microorganisms grown at optimal physiological conditions. The long-term pH stabilization with the release of ammonium carbonate offers the possibility to substantially minimize the required amount of buffer, leading to reduced osmolalities in the medium. The pH and the osmolality were thus maintained within the physiological optima of *E. coli*.

The multi-component release offers the flexibility to easily customize cultivation conditions to the requirements of a biological system, in terms of additional nutrient supply and pH stabilization. This improves the comparability with stirred-tank reactors, where a simultaneous supply of nitrogen source and alkaline solution for pH control is a common practice for cultivations with *E. coli* [51, 52].

## Methods

The following information is based on the previously published study of Philip et al. [29]

### Strain

*Escherichia coli* BL21 (DE3) pRhotHi-2-EcFbFP (GenBank number: ABN71355) [3] was used as model organism in all cultivations. The expression of a flavine mononucleotide based fluorescence protein is under the control of a *lac* operator [53, 54]. A weak fluorescence signal is detectable in uninduced cultivations due to leaky expression [3, 54].

### Media

*Escherichia coli* pre-cultures were grown in complex LB medium with 10 g/L peptone from casein (Carl Roth, Karlsruhe/Deutschland), 5 g/L yeast powder extract for bacteriology (Carl Roth, Karlsruhe/Deutschland) and 5 g/L NaCl. The pH-value was adjusted to pH 7.0–7.2. Cultivations were performed under the selection pressure of 50 mg/L kanamycin sulfate.

All main cultures of *E.* *coli* were performed with Wilms-MOPS mineral medium [22, 55] with a basic solution consisting of 6.98 g/L (NH_4_)_2_SO_4_, 3 g/L K_2_HPO_4_, 2 g/L Na_2_SO_4_ and 41.85 g/L (*N*-morpholino)-propane sulfonic acid (200 mM MOPS). The basic solution was modified in certain experiments by varying the concentration of (NH_4_)_2_SO_4_ between 6.98 g/L and 20 g/L or reducing the MOPS concentration to one-fourth to 50 mM. The pH-value was adjusted to 7.5 with NaOH. Before each experiment, the basic solution was supplemented with 0.5 g/L MgSO_4_·7H_2_O, 0.01 g/L thiamine hydrochloride, 1 mL/L trace element solution [0.54 mg/L ZnSO_4_·7H_2_O, 0.48 mg/L CuSO_4_·5H_2_O, 0.3 mg/L MnSO_4_·H_2_O, 0.54 mg/L CoCl_2_·6H_2_O, 41.76 mg/L FeCl_3_·6H_2_O, 1.98 mg/L CaCl_2_·2H_2_O, 33.4 mg/L Na_2_EDTA (Titriplex III)] and 50 mg/L kanamycin sulfate to maintain selection pressure.

For batch cultivations, the culture medium was supplemented with a glucose solution to a final concentration between 10 and 30 g/L glucose. For fed-batch cultivations, the culture medium was complemented instead with sterile distilled water to maintain the same composition of the other medium components. No glucose was provided in the culture medium but was supplied only through the reservoir system.

Before each experiment, the reservoir solution was freshly prepared from stock solutions. A 800 g/L glucose stock was used to prepare initial glucose concentrations of up to 750 g/L in the reservoir. A 250 g/L ammonium stock was used to supplement optionally the glucose solution with ammonium carbonate in the concentration range of 5–60 g/L. For experiments with 750 g/L initial glucose concentration in the reservoir, the necessary amount of ammonium carbonate had to be directly dissolved as salt in the glucose solution. For screening experiments with varying ammonium carbonate concentration in the reservoir, 17.14 g/L dipotassium phosphate was added to circumvent a possible phosphate limitation. In a separate experiment, it was shown that the presence of dipotassium phosphate did not show any significant alteration of the OTR progress (Additional file 8) and hence the changes of the abrupt OTR increases in the fed-batch phase could be correlated to the depletion of ammonium. A sufficient amount of phosphate was in the culture medium at the end of the cultivation so that the complementation with dipotassium phosphate was omitted in further experiments. The total reservoir volume was always 2 mL and blue dextran was added to a final concentration of 1 or 2 g/L. The dye with a high molecular weight (mol wt. 2,000,000, SigmaAldrich, Steinheim am Albuch/Germany) was used to detect leakage of the reservoir through absorbance measurements of the supernatant of the culture medium at 615 nm.

### Cultivation conditions

For cultivations, orbital shakers (LS-X, Kühner, Birsfelden/Switzerland) were used at 350 rpm and 50 mm shaking diameter at 37 °C. The in-house built RAMOS device enabled the quasi-continuous monitoring of the respiration activity of cultivations. Commercial versions of the RAMOS device can be obtained from Kühner AG (Biersfelden/Switzerland) or HiTec Zang GmbH (Herzogenrath, Germany). Duplicate cultivations in offline batch and fed-batch shake flasks were conducted parallelly when offline analysis was required.

For strain maintenance, cryo cultures were prepared with *E. coli* cultures grown in LB medium until mid-exponential phase. After cell harvest, 750 µL of culture medium was mixed with 250 µL of 80% (w/w) glycerol, as cryoprotectant and immediately frozen at − 80 °C. Preculture cultivations were conducted with an initial culture medium volume of 11.5 mL LB medium and 1 mL cryo culture and grown until mid-exponential phase. Cells were harvested and centrifuged at 4000 rpm and 4 °C for 15 min. The main culture medium was used for cell pellet resuspension and the master mix of the main culture was inoculated at an OD_600_ of 0.5. The initial culture medium volume of all main cultivations was 10 mL.

### Preparation of the membrane-based fed-batch shake flask

In Philip et al. [29] and Additional file 1 the set-up of the membrane-based fed-batch shake flask is shown. The first step in the set-up of the membrane-based fed-batch shake flask was the preparation of the membrane tip. The membranes used for all cultivations were a cellulosic dialysis membrane (Reichelt Chemietechnik GmbH + Co., Heidelberg/Germany) with 10–20 kDa and 42 µm thickness. A circular membrane disc with the diameter of 14 mm was punched out and fixed on the front part of the membrane tip with the help of a silicone ring (inner diameter 4 mm, wall thickness 1 mm; Saint-Gobain, Charny/France). To ensure a crease-free and consistent spanning of the membrane an in-house-built apparatus was used for this purpose. The assembled membrane tips were filled with water, positioned in a semi-micro cuvette and placed in a centrifuge for 7 min at 500 rpm to test for possible leakages. The membrane tip was used further, only when the bottom of the cuvette was liquid-free. To ensure in-phase rotation with the bulk liquid in the flask, the membrane tip was loaded with a stainless-steel ring of 3.1 g and connected to the flexible tube (inner diameter 2.06 mm, wall thickness 0.8 mm; PharMed^®^ BPT, Saint-Gobain, Charny/France) of the reservoir. After tightening all connecting parts with silicone rubber rings (inner diameter 4 mm, wall thickness 1 mm; Saint-Gobain, Charny/France) the assembled reservoir was centrally placed in the shake flask. After autoclaving, the reservoir solution was pipetted into the reservoir. With the help of a desiccator evacuated with a vacuum pump (Typ 113042, LVS, Ilmenau/Germany), entrapped gas bubbles were removed. In a final step, the culture medium was then filled sterile into the shake flask, placed on a shaker and the cultivation was started.

Additional file 9a shows the cultivation results of eight parallel shake flasks prepared in one experiment. Additional file 9b shows cultivation results from five different experiments with shake flask preparations conducted at different time points. The average values and the average standard deviations were calculated for Additional file 9. The progress of the average OTR in the batch and the fed-batch phase are similar, validating a reproducible performance of the set-up. For both data sets an average standard deviation for the time period between 2 and 5 h of 3–3.6 mmol/L/h and between 10 and 15 h of 1.2–1.8 mmol/L/h was calculated. The average standard deviation at the peak area was between 6 and 6.8 mmol/L/h. Whereas the standard deviations are slightly higher for the batch phase the results for the fed-batch show a very good reproducibility. Overall, the improved reservoir set-up proves to be robust in its application and allows for a reproducible fed-batch screening in shake flasks.

### Offline sample analysis

For determination of the optical density (OD_600_), threefold measurements were conducted in standard 1 cm cuvettes at a wavelength of 600 nm in a spectrophotometer Genesys 20 (Thermo Fisher Scientific, Bonn/Germany). To remain within the linear correlation range of 0.1 < OD_600_ < 0.3 of measurement, samples were diluted with a 0.9% (w/v) sodium chloride solution. As a blank, 0.9% (w/v) sodium chloride solution was used. The threefold cell dry weight (CDW) values were obtained through centrifugation of 0.5 mL of the respective culture medium in a previously dried and weighed reaction tube at 4000 rpm and 4 °C for 20 min. For a minimum of 48 h, the cell pellets were dried at 80 °C and weighed again. For the determination of CDWs in batch cultivations, an OD-BTM correlation with a factor of 0.33 was used. The growth rates were calculated according to the method provided in the supplementary material of Sieben et al. [56].

The highly-concentrated conditions in the reservoir cause a diffusion of water from the culture medium to the reservoir. For this reason, the cell dry weight and ammonium values were corrected with a factor based on the ratio of the initial weight of culture medium at the start and at the time point of sampling. A constant density for the culture medium was assumed. All other offline and online parameters in this study depict the actually measured values, representing the current environment of cells. The glucose and acetate concentrations in the culture medium of Fig. 1 were additionally corrected.

A pH 510 pH/mV/°C meter (Eutech Instruments Europe, Nijkerk/The Netherlands) was used for measurement of the pH-value of the supernatant. To determine the osmolality, 50 µL supernatant was measured from three samples in an Osmomat 030 (Gonotec, Berlin/Germany). Prior to measurement, a two-point calibration with water and a calibration standard (300, 500 or 850 mOsmol/kg, Gonotec, Berlin/Germany) closest to the expected osmolality of the sample was performed. The ammonium concentration of the supernatant was determined using the Spectroquant test kit (Merck KGaA, Darmstadt/Germany). The measurement was performed as described in the manual of the test kit with 10 mm cuvettes (Kartell, Noviglio/Italy) in the photometer Spektroquant NOVA 60 (Merck KGaA, Darmstadt/Germany).

Glucose and acetate quantifications in duplicates were conducted with high-performance liquid chromatography (Dionex HPLC Ultimate 3000, Sunnyvale/USA), using an Organic Acid Resin HPLC pre-column (40 × 8 mm) and an Organic Acid Resin HPLC separation column (250 × 8 mm) (both from CS-Chromatography, Langerwehe/Germany). The supernatant of the samples was diluted with distilled water to a measurable range, filtered with 0.2 µm cellulose acetate filters (VWR International, Darmstadt/Germany) and stored at − 20 °C until measurement. The measurement was performed at a flow rate of 0.8 mL/min with 5 mM sulphuric acid solution as the eluent. The temperature was set to 60 °C. The refractometric index for the detection of glucose and acetate was determined with a Shodex RI-101 detector (Techlab, Erkerode/Germany). Subsequent analysis and peak calculations were conducted using the Chromeleon software version 6.8 (Dionex Softron, Germering/Germany).

The quantification of blue dextran, for detection of leakages, was performed photometrically with a 615 nm measurement of the supernatant in the Synergy4 microtiter plate reader (Xenon flashlight, intensity 100, BioTek, Winooski/USA). Four times 200 µL of supernatant samples were measured in black 96 well microtiter plates with clear bottoms (BD Falcon TC-treated Black/Clear, Corning Incorporated, Tewksbury/United States). For data analyzation, the software Gen5 1.07 (BioTek) was used.

## Additional files

**Additional file 1.** Set-up of the membrane-based fed-batch shake flask. **(a)** The offline and **(b)** The online membrane-based fed-batch shake flask.

**Additional file 2.** Depiction of glucose amounts in the reservoir over the cultivation time. Cultivation conditions: Wilms-MOPS-mineral medium (culture medium: 200 mM MOPS with 7 g/L initial ammonium sulfate, initial reservoir glucose concentrations: 250/375/500/750 g/L, blue dextran concentration: 1 g/L, temperature: 37 °C, shaking frequency: 350 rpm, shaking diameter: 50 mm, initial culture medium volume: 10 mL, inoculation OD_600_: 0.5, reservoir filling volume: 2 mL, dialysis membrane: Reichelt 10-20 kDa, membrane area: 18.1 mm^2^; Strain: *E. coli* BL21 (DE3) pRhotHi-2-EcFbFP.

**Additional file 3.** Comparison of calculated and measured values at varied initial reservoir glucose concentrations with the membrane-based fed-batch shake flask. Depiction of **(a) + (b)** Measured OTR; **(c) + (d)** Measured and calculated amount of metabolized glucose; **(e) + (f)** Measured and calculated amount of cell dry weight; **(g) + (h)** Measured and calculated amount of metabolized ammonium and accumulated glucose. For calculations the stoichiometric equation C_6_H_12_O_6_ + 0.57 NH_3_ + 2.34 O_2_ → 3.38 CH_1.7_O_0.43_N_0.17_ + 2.62 CO_2_ + 3.99 H_2_O was used. Calculations until the abrupt OTR increases are based on measured total oxygen consumed values, calculations after the abrupt OTR increase are based on the calculated glucose release; Cultivation conditions: Wilms-MOPS-mineral medium (culture medium: 200 mM MOPS with 7 g/L initial ammonium sulfate, reservoir: 250/375/500/750 g/L glucose, blue dextran concentration: 1 g/L), temperature: 37 °C, shaking frequency: 350 rpm, shaking diameter: 50 mm, initial culture medium volume: 10 mL, inoculation OD_600_: 0.5, reservoir filling volume: 2 mL, dialysis membrane: Reichelt 10-20 kDa, membrane area: 18.1 mm^2^; Strain: *E. coli* BL21 (DE3) pRhotHi-2-EcFbFP; Concentration values have been volume-corrected.

**Additional file 4.** Depiction of ammonium concentrations in the culture medium and reservoir of the multi-component fed-batch cultivations at varied initial glucose concentrations and 40 g/L ammonium carbonate in the reservoir. Depiction of **(a)** Oxygen transfer rate; **(b)** Ammonium concentration in the culture medium; **(c)** Ammonium concentration in the reservoir; Cultivation conditions: Wilms-MOPS-mineral medium (culture medium: 200 mM MOPS with 7 g/L initial ammonium sulfate, initial reservoir glucose concentrations: 250/375/500/750 g/L, 40 g/L ammonium carbonate, blue dextran concentration: 1 g/L), temperature: 37 °C, shaking frequency: 350 rpm, shaking diameter: 50 mm, initial culture medium volume: 10 mL, inoculation OD_600_: 0.5, reservoir filling volume: 2 mL, dialysis membrane: Reichelt 10-20 kDa, membrane area: 18.1 mm^2^; Strain: *E. coli* BL21 (DE3) pRhotHi-2-EcFbFP; Concentration values have been volume-corrected.

**Additional file 5.** Depiction of cultivations with 750 g/L glucose and 40 g/L ammonium carbonate in the reservoir for the multi-component release with the membrane-based fed-batch shake flask. Cultivation conditions: Wilms-MOPS-mineral medium (culture medium: 200 mM MOPS with 7 g/L initial ammonium sulfate in culture medium, reservoir: 750 g/L glucose, 40 g/L ammonium carbonate concentration and blue dextran concentration: 1 g/L), temperature: 37 °C, shaking frequency: 350 rpm, shaking diameter: 50 mm, initial culture medium volume: 10 mL, inoculation OD_600_: 0.5, reservoir filling volume: 2 mL, dialysis membrane: Reichelt 10-20 kDa, membrane area: 18.1 mm^2^; Strain: *E. coli* BL21 (DE3) pRhotHi-2-EcFbFP; The arrow points to the occurrence of an abrupt OTR increase.

**Additional file 6.** Screening for adequate ammonium carbonate concentrations in addition to 750 g/L glucose in the reservoir for the multi-component release with the membrane-based fed-batch shake flask. Cultivation conditions: Wilms-MOPS-mineral medium (culture medium: 200 mM MOPS with 7 g/L initial ammonium sulfate in culture medium, reservoir: 750 g/L glucose, varied ammonium carbonate concentration, 17.14 g/L K_2_HPO_4_ and blue dextran concentration: 1 g/L), temperature: 37 °C, shaking frequency: 350 rpm, shaking diameter: 50 mm, initial culture medium volume: 10 mL, inoculation OD_600_: 0.5, reservoir filling volume: 2 mL, dialysis membrane: Reichelt 10-20 kDa, membrane area: 18.1 mm^2^; Strain: *E. coli* BL21 (DE3) pRhotHi-2-EcFbFP; Concentration values have been volume-corrected; The arrows point to the occurrence of abrupt OTR increases.

**Additional file 7.** Uncorrected and corrected cell dry weight results for multicomponent fed-batch cultivations. **(a)** Direct cell dry weight measurements of culture medium; **(b)** Cell dry weight measurements corrected for evaporation and water back-diffusion; Cultivation conditions: Wilms-MOPS-mineral medium (culture medium: 200 mM MOPS with 7 g/L initial ammonium sulfate, reservoir: 250/375/500/750 g/L glucose, 40 g/L ammonium carbonate, blue dextran concentration: 1 g/L), temperature: 37 °C, shaking frequency: 350 rpm, shaking diameter: 50 mm, initial culture medium volume: 10 mL, inoculation OD_600_: 0.5, reservoir filling volume: 2 mL, dialysis membrane: Reichelt 10-20 kDa, membrane area: 18.1 mm^2^; Strain: *E. coli* BL21 (DE3) pRhotHi-2-EcFbFP.

**Additional file 8.** Effect of dipotassium phosphate concentration variation on OTR in cultivations with additional glucose and ammonium carbonate in the reservoir. Cultivation conditions: Wilms-MOPS-mineral medium (culture medium: 200 mM MOPS with 7 g/L initial ammonium sulfate, reservoir: 500 g/L glucose, 40 g/L ammonium carbonate, varied dipotassium phosphate concentration, blue dextran concentration: 1 g/L), temperature: 37 °C, shaking frequency: 350 rpm, shaking diameter: 50 mm, initial culture medium volume: 10 mL, inoculation OD_600_: 0.5, reservoir filling volume: 2 mL, dialysis membrane: Reichelt 10-20 kDa, membrane area: 18.1 mm^2^; Strain: *E. coli* BL21 (DE3) pRhotHi-2-EcFbFP.

**Additional file 9.** Average values and standard deviations for validation of reproducibility. Depiction of **(a**) Eight replicates within one experiment; **(b)** Five replicates from independent experiments; Cultivation conditions: Wilms-MOPS-mineral medium (culture medium: 200 mM MOPS with 7 g/L initial ammonium sulfate, initial reservoir glucose concentrations: 250 g/L, blue dextran concentration: 4 g/L, temperature: 37 °C, shaking frequency: 350 rpm, shaking diameter: 50 mm, initial culture medium volume: 10 mL, inoculation OD_600_: 0.5, reservoir filling volume: 2 mL, dialysis membrane: Reichelt 10-20 kDa, membrane area: 18.1 mm^2^; Strain: *E. coli* BL21 (DE3) pRhotHi-2-EcFbFP.

## Abbreviations

CDW: cell dry weight
*E. coli*: *Escherichia coli*
EcFbFP: *E. coli* codon bias-optimized flavin-mononucleotide-based fluorescent reporter protein
HPLC: high-performance liquid chromatography
MOPS: (*N*-morpholino)-propane sulfonic acid
MWCO: molecular weight cut-off
OD_600_: optical density at the wavelength of 600 nm
Osm: osmolality
OTR: oxygen transfer rate
RAMOS: Respiration Activity MOnitoring System
Rpm: rotations per minute
COT: cumulative oxygen transfer
