# Supplementary figures and images for: Parallel substrate supply and pH stabilization for optimal screening of E. coli with the membrane-based fed-batch shake flask
Source: Microb Cell Fact. 2018 May 9;17:69. doi: 10.1186/s12934-018-0917-8 (PMC5941677; doi:10.1186/s12934-018-0917-8)

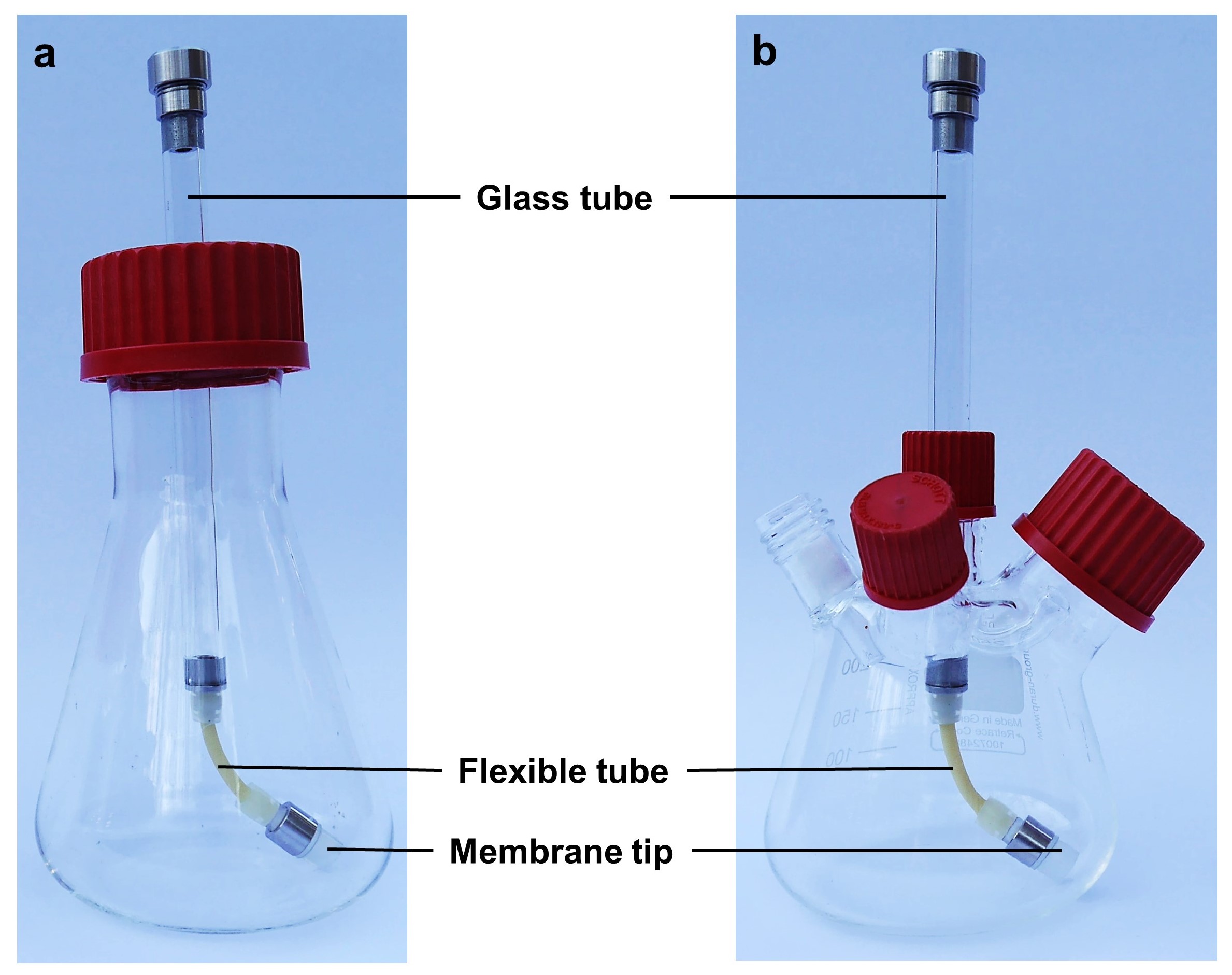

Supplement: Supplementary file 1 — Additional file 1. Set-up of the membrane-based fed-batch shake flask. (a) The offline and (b) The online membrane-based fed-batch shake flask. [file 12934_2018_917_MOESM1_ESM.jpg]
